# Neomedia Repair of the Valsalva Sinus in the Treatment of Acute Type-A Aortic Dissection: Long-term Effectiveness and a Case of Pathology

**DOI:** 10.3400/avd.oa.20-00113

**Published:** 2020-09-25

**Authors:** Nobuhisa Ohno, Toshi Maeda, Otohime Kato, Hirofumi Sato, Go Ueno, Kosuke Yoshizawa

**Affiliations:** 1 Department of Cardiovascular Surgery, Amagasaki General Medical Center

**Keywords:** aortic dissection, aortic root, neomedia, polyester fabric, pathology

## Abstract

Although numerous surgical techniques are employed to treat acute Stanford type A aortic dissection (ATAAD), controversy remains over which is the best procedure for aortic root reconstruction. Among the various techniques utilized, neomedia repair is considered to be more promising than adhesive-only repair for the treatment of a dissected aortic root. We experienced a series of neomedia sinus Valsalva repair using woven polyester fabric, and evaluated the aortic root diameter by computed tomography and severity of aortic valve insufficiency by transthoracic echocardiography. The aortic root diameter was well preserved with no progress of aortic valve insufficiency in the long-term period. Furthermore, we found that the fabric looked functioning new media in the findings obtained from the pathological examination of a neomedia repaired aortic wall sample that was obtained by chance from a patient during valve replacement surgery performed 10 years after aortic reconstruction for ATAAD. Neomedia repair using woven polyester fabric for ATAAD might facilitate the long-term durability of the surgically treated aortic root. (This is a translation of J Jpn Coll Angiol 2019; 59: 37–43.)

## Introduction

The basic treatment method for acute type A aortic dissection (ATA AD), excluding cases with acute thrombotic occlusion, is emergency surgery,^1)^ which is generally performed with either ascending aorta replacement (AAR) or total arch replacement (TAR). For patients with severe preoperative condition, saving the patient’s life is the first priority; however, recent advancements in extracorporeal circulation, brain and heart preservation, and revascularization methods^2,3)^ have improved the outcomes of the initial treatment, and the improvement of long-term prognosis should always be kept in mind. Long-term prognosis should be considered for the remaining aortic root and distal aorta separately; however, the prognosis for the aortic root is affected by the enlargement of the sinus of Valsalva caused by recanalization and associated aortic insufficiency (AI).^4)^ To stabilize the dissected sinus of Valsalva, we performed a series of neomedia repairs using polyester fabric as artificial media inserted into the dissected cavity, which was followed up for as long as 10 years. We presently report that we examined the long-term outcomes and obtained very interesting findings in one patient who underwent repeat surgery.

## Subjects and Methods

Among 23 consecutive patients with ATA AD treated between November 2008 and December 2012, we included 18 patients who underwent neomedia repair of dissected sinus of Valsalva with aortic root preservation. All patients underwent emergency surgery immediately after ambulance transportation to our hospital for acute aortic dissection. In patients who were directly transported by ambulance, diagnosis could be obtained by contrast-enhanced computed tomography (CT) at our hospital, and therefore, when the previous physician performed the CT, the treatment policy was determined based on such information.

Surgery was performed using a median sternotomy approach under general anesthesia with the patient in the supine position. For the extracorporeal circulation blood delivery site, when there is an undissected area on the lesser curvature side of the ascending aorta, that area is selected first, and an 18 Fr blood supply cannula for percutaneous cardiopulmonary support (PCPS) was inserted using the Seldinger technique. When the dissection extended to the lesser curvature side of the ascending aorta, a supply route was established with an end-to-side anastomosis performed using the femoral artery in young patients with mild arteriosclerotic lesions and using an 8-mm prosthetic graft from the right axillary artery for elderly patients and those with the dissection extending to the femoral artery. When malperfusion is observed in the four limbs, these techniques were combined, and at the start of the extracorporeal circulation, we endeavored to correct the malperfusion as much as possible. The extracorporeal circulation was commenced by taking the blood from two vessels, that is, the superior and inferior vena cavae, after which a retrograde coronary perfusion cannula and left ventricle vent were inserted. A cooling period of at least 30 min was left, and after confirming that both the temperature of the blood and tympanic temperature were below 26°C, circulatory arrest was determined. Cerebroprotection was performed by retrograde cerebral perfusion until confirmation of the intra-arch findings, and at the time of determining the surgical procedure, we switched to antegrade selective cerebral perfusion. For the myocardial protection, retrograde coronary perfusion-based antegrade selective coronary perfusion was additionally performed. When a dissection entry was present in the ascending aorta and there was no expansion of the dissection into the arch portion of the aorta, AAR was performed, and when there was an expansion into the aortic arch, with a dissection entry in the arch portion, or in cases of DeBakey type III retrograde dissection, TAR was performed. For all prosthetic grafts, a single-branch or 4-branch polyester graft was used.

After performing distal anastomosis, proximal anastomosis followed for AAR and distal anastomosis by stepwise technique for TAR. Anastomosis was performed on the three branches of the arch in a distal-to-proximal order for TAR. Following the completion of distal anastomosis, the circulation of the lower half of the body was recommenced from the lateral branch of the prosthetic graft, and rewarming was slowly started.

**Figure 1:**
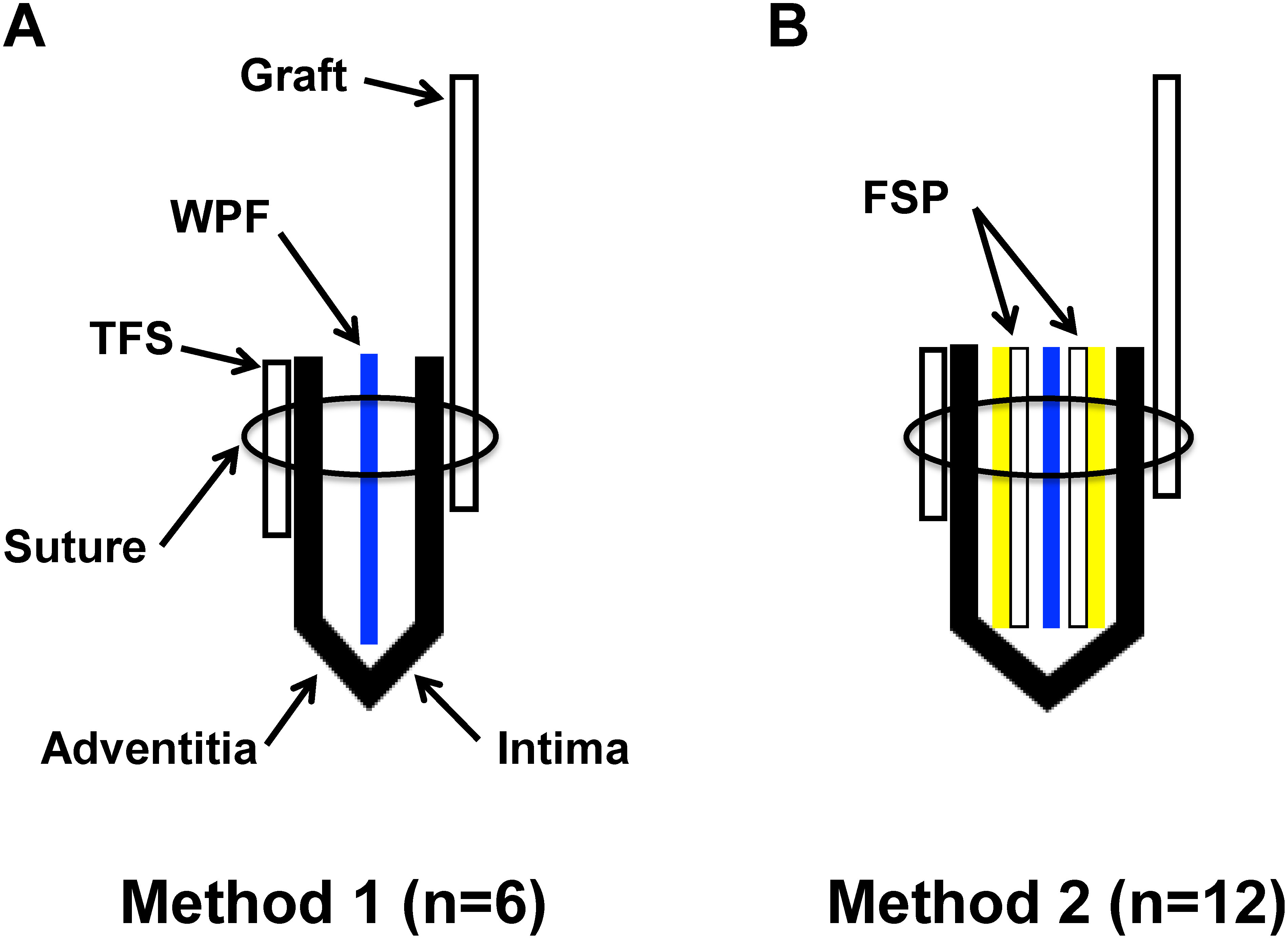
Fig. 1 Schema of the neomedia repair technique.

**Figure 2:**
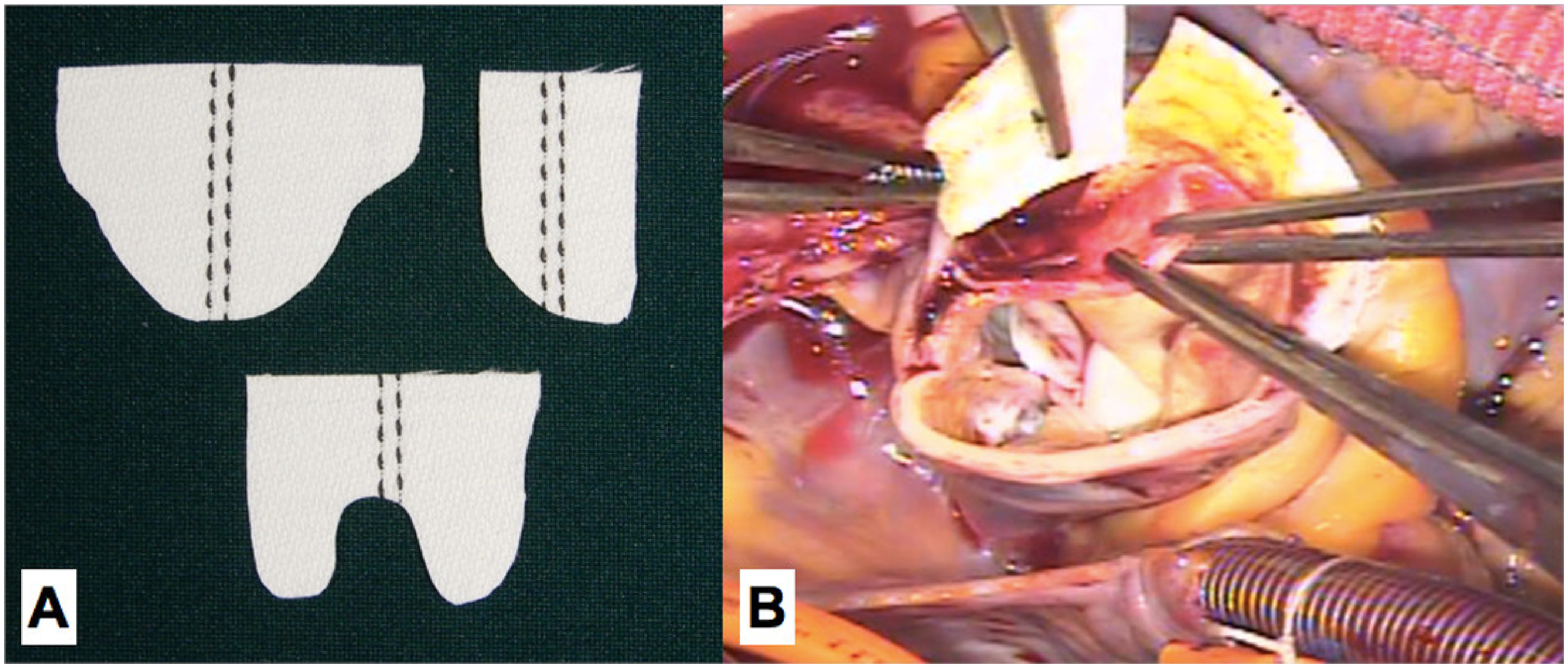
Fig. 2 Photograph of prepared neomedias and an operative procedure.

Neomedia repair was performed on the sinus of Valsalva immediately before the proximal anastomosis. In this series, two types of methods were used. The first method involved inserting polyester fabric (Hemashield Woven Double Velour Fabric; Getinge, Dubai, UAE) cut to the shape of the dissected sinus of Valsalva into the dissected cavity and pasting with a fibrin glue (Beriplast; CSL Behring, King of Prussia, PA, USA) (**Fig. 1A**). To obtain uniform adhesive strength, the polyester fabric was soaked in A solution of fibrin glue, and after inserting into the dissected cavity, B solution was added; however, when the tissue is wet, adhesion cannot be achieved, and on occasion, fibrin glue needs to be added. Therefore, method 2 was improved to be faster and to obtain a more secure adhesion. That is, the surface of the collagen layer of two sheets of a fibrin sealant patch (TachoSil; CSL Behring, King of Prussia, PA, USA) cut to the same shape of the polyester fabric was pasted to the back of the polyester fabric to create a patch consisting of a fibrinogen/thrombin layer with both surfaces matching the shape of the dissected sinus of Valsalva. This was inserted into the dissected cavity and pressure was applied manually to repair the dissected cavity (**Fig. 1B**). This method enabled a stable adhesive strength to be obtained. In both methods, the neomedia was trimmed to match the shape of the dissected cavity, and when there were several dissections extending over the sinus of Valsalva, the shape of each dissection of the sinus of Valsalva was copied and reconstructed separately (**Fig. 2**). After lifting each commissure of the aortic valve using a 4-0 monofilament thread with felt, a belt-like felt was wrapped around the circumference of the aorta, and a prosthetic graft inserted directly over the commissure was anastomosed using a 4-0 monofilament thread with whole-circumferential U-shaped interrupted sutures.

(**A**) Method 1 (n=6); A geometrically tailored WPF (blue) is inserted into the dissected Valsalva sinuses with the aid of fibrin glue to adhere dissected layers. The prosthetic graft is anastomosed with external TFS reinforcement. (**B**) Method 2 (n=12); Two FSPs tailored as the same shape as the WPF (blue) are put on both sides of the WPF with the aid of fibrin glue to make the composite patch. The dry fibrinogen/thrombin layers (yellow) face outward as an adhesive surface. The composite patch is inserted into the dissected Valsalva sinuses, and strong bonding between the adventitia and the intima is obtained. The prosthetic graft is anastomosed with the same fashion as method 1. WPF: woven polyester fabric; TFS: teflon felt strip; FSP: fibrin sealant patch

(**A**) Various forms of neomedia are tailored to fit dissected Valsalva sinuses. (**B**) Dissected left to right Valsalva sinuses are repaired by method 2. Two pieces of neomedia prepared with FSP are inserted in the sinuses.FSP: fibrin sealant patch

The distal anastomosis was performed with the stump formation method; however, for the distal anastomosis in TAR, considering that the inserted elephant trunk contributes to the stabilization of the intima, we concurrently used the adventitial inversion technique and performed anastomosis of the prosthetic graft with whole-circumferential U-shaped interrupted and uninterrupted sutures using a 4-0 monofilament thread. For the distal anastomosis in AAR, after the stump formation using the same neomedia repair method as proximal anastomosis, we similarly used whole-circumferential U-shaped interrupted sutures.

Prior to hospital discharge, all patients underwent contrast-enhanced CT with transthoracic echocardiography and received postoperative follow-up on an outpatient basis at our department. As a rule, plain CT and transthoracic echocardiography were performed each year. The maximum diameters of the aortic root obtained before discharge and at the final follow-up were measured and compared using an image analysis software (Centricity PACS Universal Viewer, ver.6.0; GE Healthcare, Chicago, IL, USA). Transthoracic echocardiography was performed in the physiological examination room, and the presence or absence and degree of AI were compared before discharge and at the final examination. AI was evaluated according to the test report standard. When trimming the aortic wall in one patient who underwent long-term repeat surgery, a small sample of the neomedia repair site was taken, and histopathological diagnosis was made using hematoxylin eosin (HE) staining. Continuous variables are presented as mean±standard deviation, and a survival curve was created with the Kaplan–Meier method using JMP (SAS Institute Inc., Cary, NC, USA). This study was performed with the informed consent of the patient and their family members and with the approval of the ethical review board of Amagasaki General Medical Center, Hyogo (approval number: 31-11).

## Results

The subject sample included 18 patients with a mean age of 64.5±12.2 years (range, 45–88 years), consisting of 9 male and 9 female patients. Preoperative cardiac tamponade was observed in five patients, and cardiopulmonary resuscitation was performed in one patient. Cerebral malperfusion was observed in two patients, and malperfusion of the lower limbs was observed in two patients. Dissection entries were located in the ascending aorta in 13 patients and in the aortic arch in two patients, with DeBakey type III retrograde dissection in three patients. AIs measured preoperatively using transthoracic and transesophageal echocardiography were mild in four patients and moderate or greater in two patients. One patient underwent maintenance dialysis for chronic renal failure (**Table 1**). AAR was performed in 13 patients, and TAR in 4 patients, with partial arch replacement performed to reduce the operative duration in one patient who underwent cardiopulmonary resuscitation. Concurrent procedures included coronary artery bypass in one patient. Among patients who underwent TAR, type 0 congenital bicuspid aortic valve was observed in one patient; however, the aortic valve was preserved because there was no functional problem. The mean operative duration was 401±76 min, the mean extracorporeal circulation duration was 228±50 min, the mean myocardial ischemia duration was 129±38 min, and the duration of circulatory arrest of the lower half of the body was 65±37 min (**Table 2**).

**Table 1:** Table 1 Preoperative patient characteristics

Variables	n=18
Age, years	65±12
Female (%)	9 (50)
Cardiac tamponade	5
Aortic regurgitation	
Mild	4
Moderate	2
Malperfusion	
Brain	2
Limb	2
Chronic renal failure	1
DeBakey type III retrograde dissection	3
CPR	1

CPR: cardiopulmonary resuscitation

**Table 2:** Table 2 Operative data and finding

Variables	n=18
Operation time, minutes	401±76
CPB time, minutes	228±50
Cardiac ischemic time, minutes	129±38
Circulatory arrest time, minutes	65±37
Replacement of the aorta	
Ascending aorta	13
Ascending aorta+partial arch	1
Ascending aorta+total arch	4
Concomitant procedure	
CABG	1
Operative finding	
Bicuspid aortic valve	1

CPB: cardiopulmonary bypass; CABG: coronary artery bypass grafting

**Figure 3:**
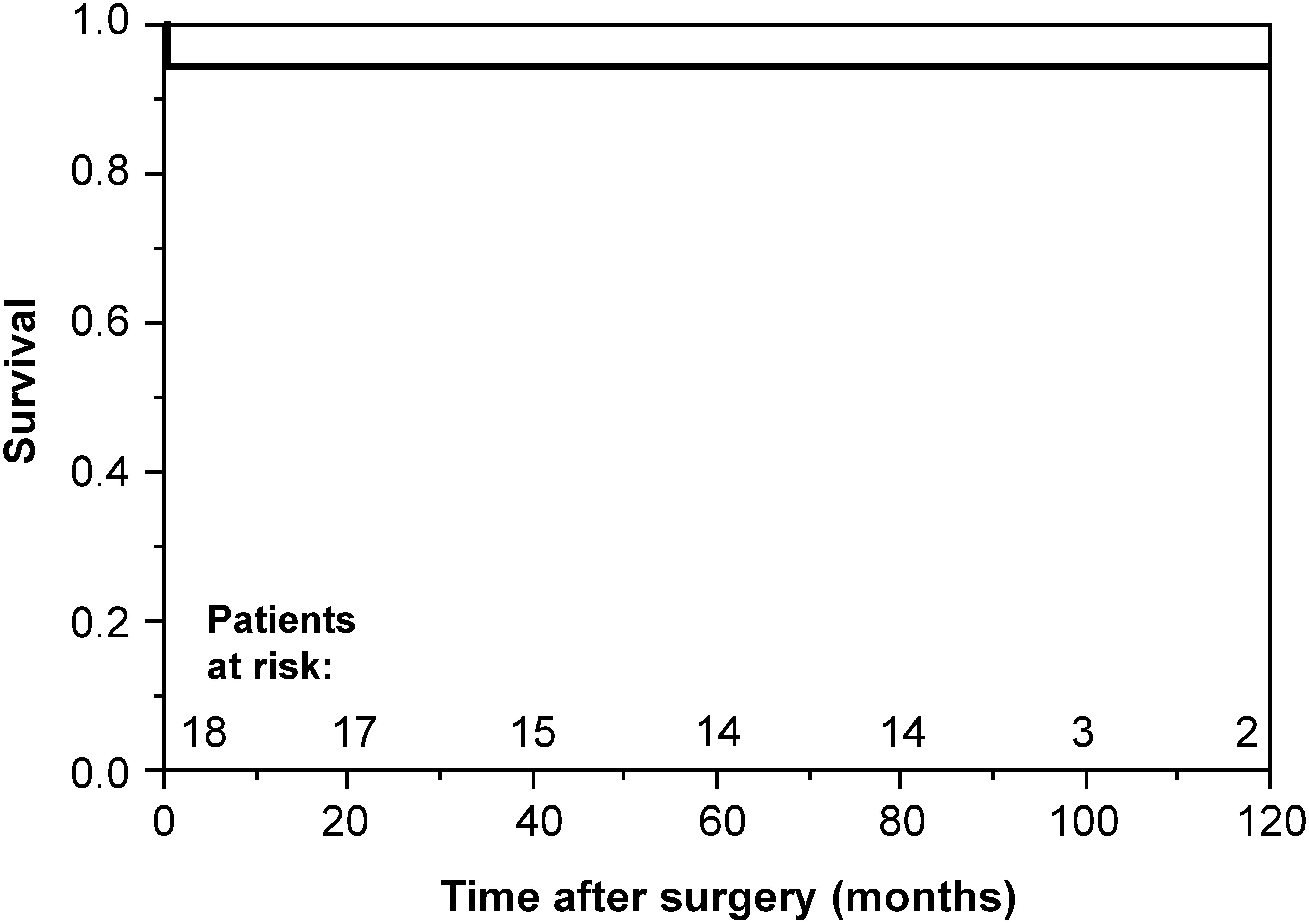
Fig. 3 Postoperative survival including hospital death.

**Figure 4:**
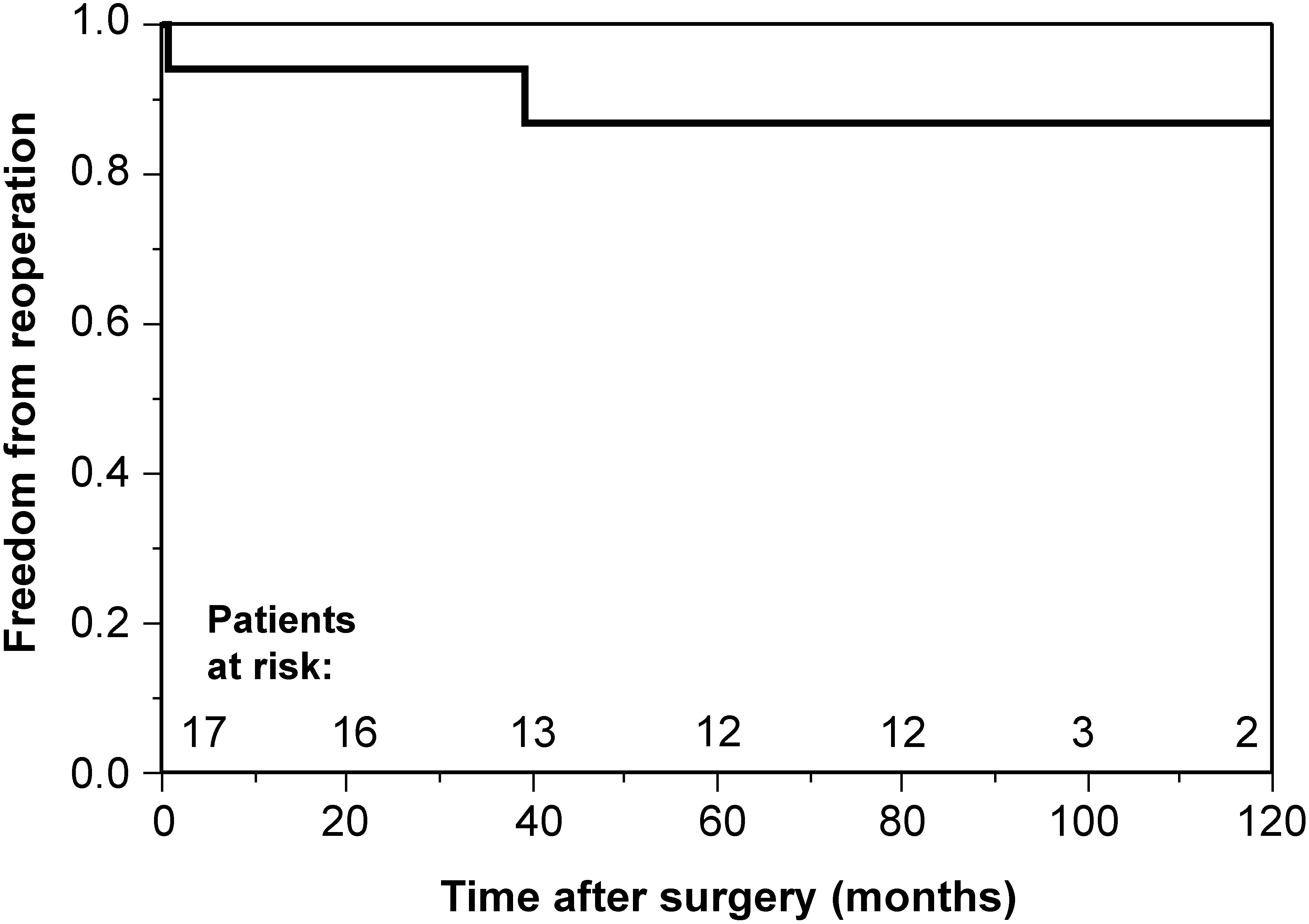
Fig. 4 Freedom from aortic reoperation.

**Figure 5:**
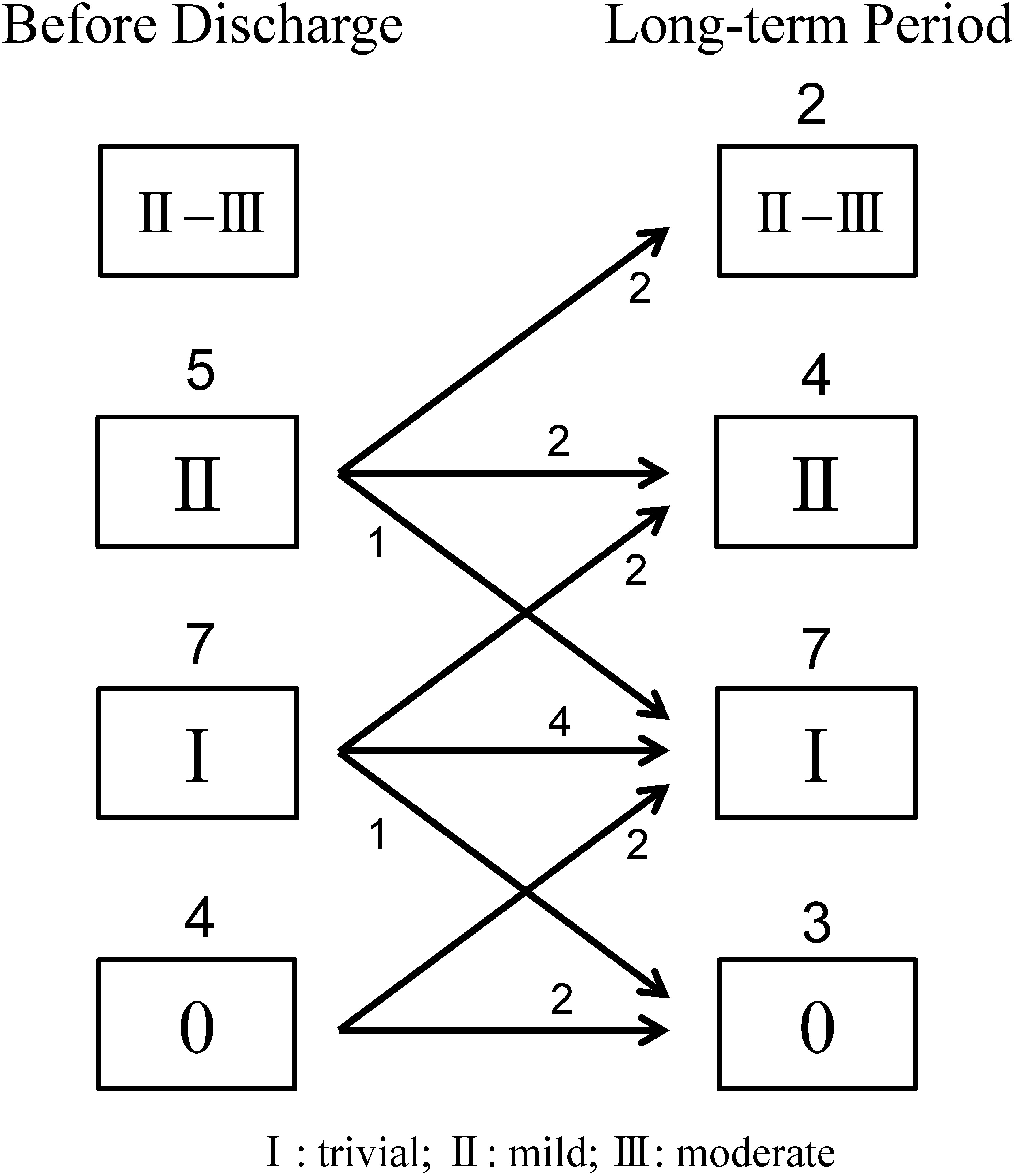
Fig. 5 Postoperative follow-up of aortic regurgitation.

The patient who underwent preoperative cardiopulmonary resuscitation died due to total cerebral ischemia one week after surgery. The other 17 patients were discharged in recovery without any major complications. Postoperative long-term interventions for the aorta included replacement of the thoracoabdominal aorta due to persistent dissection and thoracoabdominal aorta enlargement, with abdominal debranching and thoracic endovascular aortic repair (TEVAR) in one patient. Cardiac interventions included mitral annuloplasty for mitral insufficiency caused by rupture of the chordae tendineae in one patient, and in the patient with congenital bicuspid aortic valve, AI progressed, and 10 years later, aortic valve replacement was performed. In this patient, calcification of the right coronary cusp in the undissected sinus slowly progressed, and rupture of the same site led to AI. No patient required intervention for the aortic root (**Table 3**). Three patients dropped out during the follow-up due to difficulty in visiting the hospital because of dementia; however, the remaining 14 patients still continue to visit the outpatient services. As a result, the postoperative survival rates at 1, 5, and 10 years were 94%, 94%, and 94%, respectively (**Fig. 3**), and the rates of avoiding repeat surgery of the aorta including the distal aorta were 94%, 87%, and 87%, respectively (**Fig. 4**). The maximum short axis diameter of the aortic root on CT did not change (35.8±3.2 mm at discharge vs. 34.5±4.8 mm on the final CT) (**Table 4**). Changes in AI on transthoracic echocardiography from discharge to the long term are presented in the figure; however, in most patients, the status at discharge was maintained, and there were no patients observed in whom AI would pose a clinical problem (**Fig. 5**).

**Table 3:** Table 3 Postoperative events

Variables	n=18
Hospital death*	1
Aortic reintervention	
TEVAR	1
Replacement of the thoracoabdominal aorta	1
Replacement of the abdominal aorta	1
Aortic valve replacement**	1
Mitral valve repair	1

TEVAR: thoracic endovascular aortic repair * cardiopulmonary resuscitation, ** bicuspid aortic valve

**Table 4:** Table 4 Long-term follow-up of the aortic root diameter

	Before discharge	Long-term*
Root diameter, millimeter	35.8±3.2	34.5±4.8

* 62±34 months (31～120; median 68)

**Figure 6:**
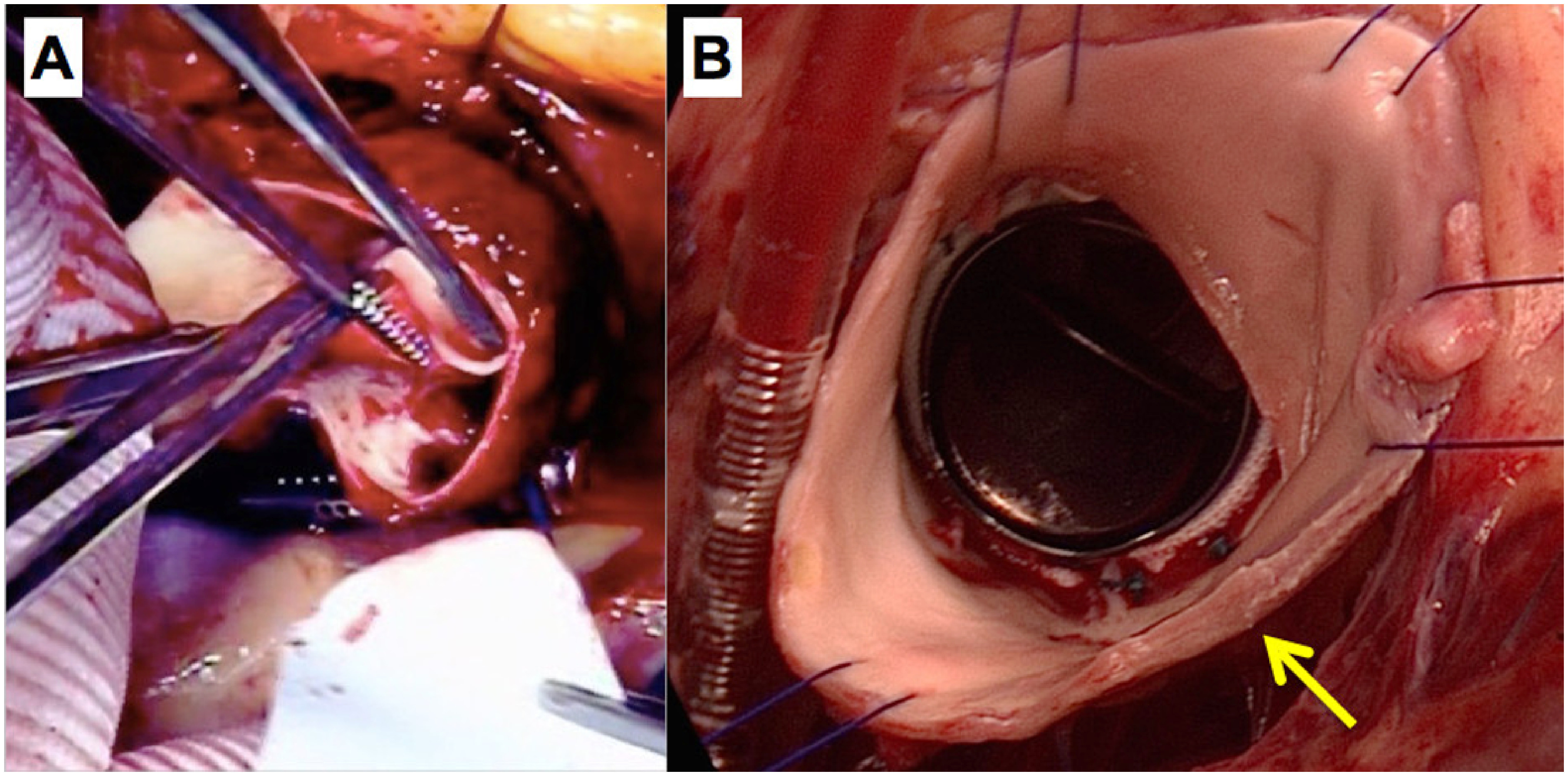
Fig. 6 Intraoperative views of the first operation and the second aortic valve replacement for a patient of degenerated bicuspid aortic valve.

**Figure 7:**
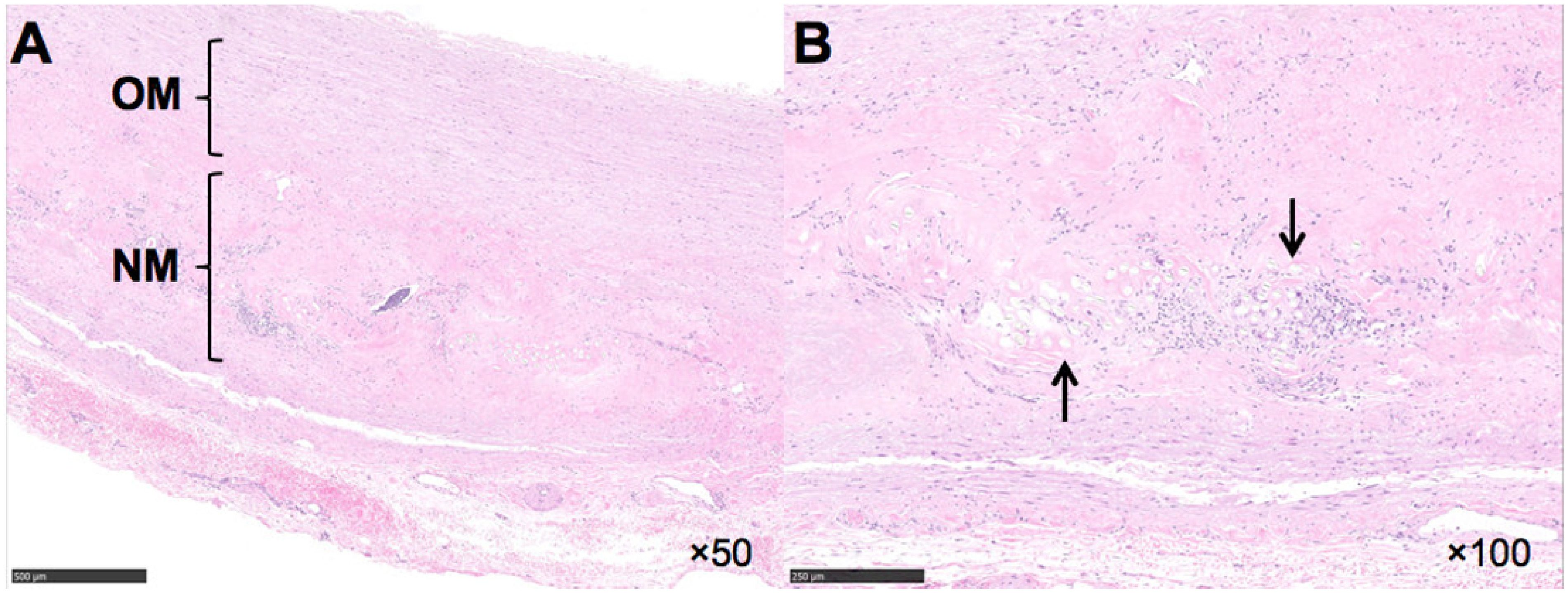
Fig. 7 Pathological view of the neomedia repaired aortic wall derived from a patient who had the aortic valve replacement for the degenerated bicuspid aortic valve.

For the patient with bicuspid aortic valve who required repeat surgery in the long term, the initial surgery findings (**Fig. 6A**), repeat surgery findings (**Fig. 6B**), and histopathological findings of the aortic wall (**Fig. 7**) were presented. At the time of the initial surgery, there was no enlargement of the sinus of Valsalva, and good initial adhesion was obtained by performing method 1 (**Fig. 6A**). At the time of repeat surgery, the status of the neomedia repair site was maintained and could not be macroscopically distinguished from the normal aortic wall (**Fig. 6B**). The histopathological specimen of the same site revealed that the fibers of the polyester fabric formed a new media (neomedia) that was unified with its own native media (**Fig. 7**).

(**A**) Dissected non-coronary to right-coronary sinus was repaired by method 1. (**B**) The finding showed normally healed aortic wall repaired by neomedia (yellow arrow).

(**A**) Neomedia (NM) layer situates just outside the native own media (OM) (HE, ×50). (**B**) Artificial fibers (some of them are indicated by arrows) are seen in the NM layer without active inflammation (HE, ×100). HE: hematoxylin and eosin

## Discussion

With regard to prosthetic graft replacement in ATA AD, various stump formation methods have been reported in the past. Such methods have long since started with methods for adhering the dissected cavity using an adhesive including a belt-shaped felt sandwich,^5,6)^ adventitial inversion,^7)^ gelatin–resorcin–formalin (GRF) glue, bovine serum albumin–glutaraldehyde glue (BioGlue; CryoLife, Inc., Kennesaw, GA, USA), fibrin glue (Beriplast; CSL Behring, King of Prussia, PA, USA), and fibrin sealant patch (TachoSil; CSL Behring, King of Prussia, PA, USA),^8–10)^ as well as a neomedia repair method by inserting a felt sheet^11,12)^ or polyester fabric^13,14)^ into the dissected cavity as an artificial membrane. Stump formation was originally developed to prevent bleeding from the artificial anastomosis site and to stabilize the dissected intima so as not to cause a new entry by cutting the intima at the site of anastomosis. Alternatively, the treatment of the side of the dissected aortic root requires stabilization of the dissected sinus of Valsalva and maintenance of the aortic ring morphology in addition to reinforcement of the artificial anastomosis site, which should be considered separately. Biological adhesives such as GRF and BioGlue are injected into the base of the dissected cavity in the aim of total formation of the sinus of Valsalva; however, it has been pointed out that tissue toxicity of such adhesives causes problems.^15–17)^ Neomedia repair involves the insertion of an artificial fabric tailored to the shape of the dissected sinus of Valsalva, and adhesion using either biological adhesive enables greater strength to be obtained in the sinus of Valsalva formed. For the material used, Teflon felt and polyester knitted fabrics have been reported.

Regarding the long-term prognosis of the aortic root formed, there is not a single report summarizing surgical procedures. Sabik et al.^18)^ reported that in AAR with stump formation in the aortic root using felt and aortic commissure lifting in 135 patients with acute and chronic ascending aorta dissections, the rate of avoiding repeat surgery for the aortic valve and aortic root was 96% at 5 years and 93% at 10 years. The reason for repeat surgery was enlargement of the sinus of Valsalva in two patients, infectious endocarditis in two patients, and prosthetic graft infection in one patient. Kirsch et al.^19)^ performed stump formation in 130 patients, among whom GRF was used in more than two-thirds of the patients, and they reported that repeat surgery was avoided at a rate of 75% at 5 years, 61% at 10 years, and 39% at 15 years, which might have been affected by GRF problems.

While the details underlining the cause of repeat surgery are unclear, repeat surgery includes many procedures such as aortic valve replacement, AAR, and the Bentall procedure, and it is thought that AI and proximal aortic enlargement are causes. Geirsson et al.^20)^ formed the sinus of Valsalva using neomedia and avoided repeat surgery of the aortic root at a rate of 95% at 5 years and 78% at 10 years. The primary cause of repeat surgery of the proximal aorta was AI and anastomotic false aneurysm. In this study, BioGlue was used in the middle of neomedia fixation, and the effect of which cannot be ruled out. Rylski et al.^12)^ formed the sinus of Valsalva using BioGlue and felt neomedia in 489 patients and avoided repeat surgery of the proximal side at a rate of 96% at 5 years, 92% at 10 years, and 89% at 15 years. The cause of repeat surgery was AI and anastomotic false aneurysm. Alternatively, Nakajima et al.^14)^ performed neomedia repair by fixing knitted polyester fabric with fibrin glue and reported good outcomes with repeat surgery of the aortic root avoided at a rate of 98% at 5 years and 98% at 10 years. Although not resulting in repeat surgery, residual dissection of the aortic root was observed on CT during follow-up in three patients. In all reports, repeat surgery was performed for AI, anastomotic false aneurysm, and enlargement of the sinus of Valsalva, and we believe that maintaining the residual sinus of Valsalva and strength of the aortic commissure is the key to avoiding repeat surgery.

Our method differs to reports to date in terms of the fact that a sheet of the same polyester fabric generally used for prosthetic grafts of the thoracic aorta was used as the neomedia and that in method 2, a fibrin sealant patch was used for adhesion. Polyester woven fabric is thin with moderate tension and has good operability when inserting to the back of the dissected cavity. Based on the histological findings presently obtained at the time of repeat surgery, affinity with the native tissue was also good, and therefore, we believe that it is a suitable material as artificial media. With regard to the difference between methods 1 and 2, repeat surgery was performed for the patient of method 1, and a fibrin glue was used as the bioabsorbable material, and therefore, we believe that if proper adhesion is achieved initially, long-term strength maintenance might lie in the polyester woven fabric, with similar outcomes. However, regarding the surgical technique, the fibrin sealant patch was superior in which the additional application of fibrin glue, which carries concern for embolism, was not needed, and proper adhesion could be obtained in a short time. Furthermore, it has been reported that the fibrin sealant patch is effective as an adhesive material for the dissected cavity when used alone,^9)^ and because there is very little concern of biotoxicity, it is considered useful as an adhesive crosslink.

A limitation of the present study was that it was a retrospective observational study with a small subject sample. However, given that there was neither a single case of aortic root enlargement nor repeat surgery involving the aorta, we believe that this method is an excellent surgical procedure.

## Conclusion

When performing AAR for ATA AD, we inserted a polyester woven fabric into the dissected sinus of Valsalva and performed neomedia repair using fibrin glue and a fibrin sealant patch as an adhesive crosslink. In the long-term outcomes for up to 10 years, there were no patients with enlargement of the sinus of Valsalva and progression of AI, with repeat surgery of the aorta and aortic valve related to dissection avoided at a rate of 100%. The histopathology of the patient with bicuspid valve who incidentally required repeat surgery revealed that the neomedia completely assimilated with the native tissue, and we believe that this method greatly contributes to the repair and strength maintenance of the dissected sinus of Valsalva.

## Notes

This is a translation of J Jpn Coll Angiol 2019; 59: 37–43.
